# Major Histocompatibility Complex Class II Deficiency Beyond Infancy

**DOI:** 10.1155/crii/8570051

**Published:** 2025-07-27

**Authors:** Aziza Bachir Kattra, Ibtihal Benhsaien, Asmaa Drissi Bourhanbour, Zahra Aadam, Abderrahmane Errami, Fatima Ailal, Ahmed Aziz Bousfiha, Jalila El Bakkouri

**Affiliations:** ^1^Laboratory of Clinical Immunology, Infection and Autoimmunity (LICIA), Faculty of Medicine and Pharmacy, Hassan II University of Casablanca, Casablanca, Morocco; ^2^Department of Pediatric Infectious Diseases and Clinical Immunology, Abderrahim Harouchi Mother-Child Hospital, Ibn Rochd University Hospital, Casablanca, Morocco; ^3^Immunology Laboratory, University Hospital Center Ibn Rochd, Casablanca, Morocco; ^4^Immunopathology-Immunotherapy-Immunomonitoring Laboratory, Mohammed VI University of Sciences and Health (UM6SS), Casablanca, Morocco

**Keywords:** combined immunodeficiency, HLA-DR expression, inborn errors of immunity, MHC-II deficiency, RFXANK mutation

## Abstract

Major histocompatibility complex class (MHC)-II deficiency is a rare autosomal recessive combined immunodeficiency, accounting for 4.1% of inborn errors of immunity (IEI) cases in North Africa and the Middle East. Most patients do not survive beyond the age of 10 years. The cases described in this study are rare and unusual for MHC-II deficiency. We report the cases of four unrelated patients of Moroccan origin with MHC-II deficiency. Immunophenotyping of lymphocyte subpopulations and analysis of human leukocyte antigen-DR (HLA-DR) expression were performed using flow cytometry. Genetic analysis was conducted through direct sequencing. The mean age of our patients was 18.75 years (range 16–26 years); the mean age at diagnosis was 14.07 years, and the mean age of onset of symptoms was 5.25 months. The clinical presentation is characterized by recurrent pulmonary infections with predominant bronchial dilatation and hemorrhagic rectocolitis. The diagnosis was confirmed in all patients by absence of HLA-DR expression and detection of the c.338-25_338del mutation in *RFXANK*. Three (75%) of our patients are still alive and are on monthly intravenous immunoglobulin (IVIG) therapy. It is important to consider MHC-II deficiency in the differential diagnosis of combined immunodeficiencies across all age groups. Further studies are needed to elucidate the various phenotypes associated with this condition.

## 1. Introduction

Major histocompatibility complex class II (MHC-II) deficiency is an inborn errors of immunity (IEI) affecting cellular and humoral immunity [1, 2]. This rare disease has been reported in 702 patients in North Africa and the Middle East, with a prevalence in the West estimated at 1/166,000 to 1/350,000 live births [3].

The disease is caused by mutations in one of four transcription factors, ranked by their order of discovery [4–6]. This classification includes the class II transactivator (CIITA, group A), the regulatory factor X-containing ankyrin repeats (RFXANK, group B), the fifth member of the regulatory factor family (RFX-5, group C), and the regulatory factor X-associated protein (RFXAP, group D) [7–9]. These mutations lead to altered antigen presentation by human leukocyte antigen-DR (HLA-DR), HLA-DQ, and HLA-DP molecules on antigen-presenting cells such as dendritic cells and macrophages [10–12]. This leads to early onset of severe, recurrent infections, mainly respiratory and gastrointestinal tract infections, as well as developmental delays and premature death [13]. The most common infectious agents are cytomegalovirus, herpes simplex virus (HSV), streptococcal and staphylococcal infections, Cryptosporidium, and *Pneumocystis jiroveci* [14]. The only curative treatment is hematopoietic stem cell transplantation (HSCT). Previous studies have reported transplant-free survival of less than 10 years. Prognosis is very poor, with the mean age at the time of death being 4 years [15]. MHC-II deficiency is often described in infants aged between 2 and 12 months, with an average age of 4.5 months.

This study presents exceptional cases of MHC-II deficiency in older children and adults. We describe the cases of three adolescents and a 26-year-old adult of Moroccan origin. A literature review discusses the demographic, clinical, and immunophenotypic features of similar cases.

## 2. Materials and Methods

Four patients with MHC-II deficiency were recruited from the Pediatric Department of Infectious Diseases and Clinical Immunology at Abderrahim El Harouchi Children's Hospital, Ibn Rochd University Hospital, Casablanca, Morocco. Clinical data were retrieved by chart review. All our patients underwent a complete blood cell count and HIV testing. Lymphocyte subpopulation count was performed on all study patients, using flow cytometry (BD Biosciences, San José, CA). The markers used were CD3+, CD4+, CD8+, CD19+, CD16+/CD56+, and HLA-DR.

### 2.1. Genetic Analysis

We investigate the c.338-25_338del26 mutation due to its status as a founder effect mutation in the Moroccan population [16]. To detect this mutation, we amplified the region using polymerase chain reaction followed by direct sequencing (Illumina HiSEQ, USA). The primers used were RFXANK-6-F (5-TTG GCA-GCA-CTG-GGG-ATA-G-3) and RFXANK-6-R (5-CCA-GCA-GAC-ACA-GCC-AAA-AC-3). DNA was extracted from peripheral whole blood using the phenol–chloroform method or the iPrep Pure Link gDNA Blood Kit and iPrep Instruments (Life Technologies, Thermo Fisher Scientific).

### 2.2. Statistical Analysis

Statistical analyses were performed using Microsoft Excel (Version 2019, Microsoft Corporation, Redmond, WA, USA) and GraphPad Prism (version 8.4.3, GraphPad Software, San Diego, CA, USA).

### 2.3. Ethics and Consent

Ethical approval was obtained from the Ethics Committee of the Abderrahim Harouchi Children's Hospital, Ibn Rochd University Hospital, Casablanca, Morocco (Approval Number: 2020/DoEHRSI/039). All study patients provided informed consent.

### 2.4. Literature Review

All series and case reports of MHC-II deficiency published in English or French in the literature were reviewed. Inclusion criteria were HLA-DR expression <5% and age ≥15 years. All patients who had undergone hematopoietic stem cell or umbilical cord transplants were excluded. Of the remaining studies, only three were retained, and a total of eight patients met our criteria (Table 1). The demographic, clinical, immunophenotypic, and genetic information of each patient was recorded.

## 3. Results

### 3.1. Cases Presentation

P1: I.S. is a 16-year-old boy, the youngest of four siblings. He has no indication of consanguinity and has a family history of a paternal cousin who died at 4 months of age due to respiratory distress.

His medical history began at 11 months when he was hospitalized for recurrent respiratory infections. Then, at the age of 3 years he was monitored for meningitis. Later at 9 years old, he was experiencing a chronic bronchorrhea associated to bilateral recurrent sinusitis. He also presented with watery diarrhea and abdominal pain, which was managed with a diet excluding cow's milk protein. Additionally, he showed failure to thrive throughout his childhood.

**Figure 1:**
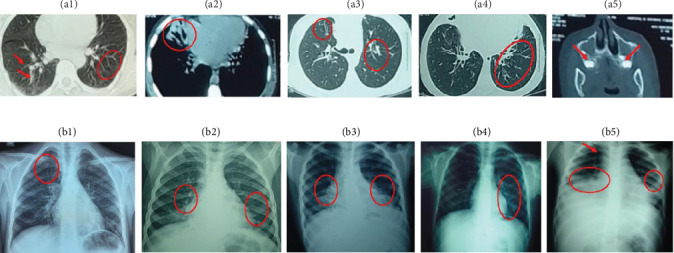
Radiographic and CT imaging of patients with MHC-II deficiency. (A) Chest CT scan images showing bronchiectasis in patient P1 (a1, a2) and patient P2 (a3, a4). Image a5 shows sinusitis in patient P2. (B) Chest X-ray images showing clarity on the upper left lobe with a mild distention in patient P1 (b1) and bronchiectasis associated with lobar condensation and mediastinal adenopathy in patient P2 (b2, b3, b4). Image b5 (P2) shows right lower lobe and left lower lobe condensation with significant thoracic distention, revealing pneumonia associated to mediastinal enlargement.

**Figure 2:**
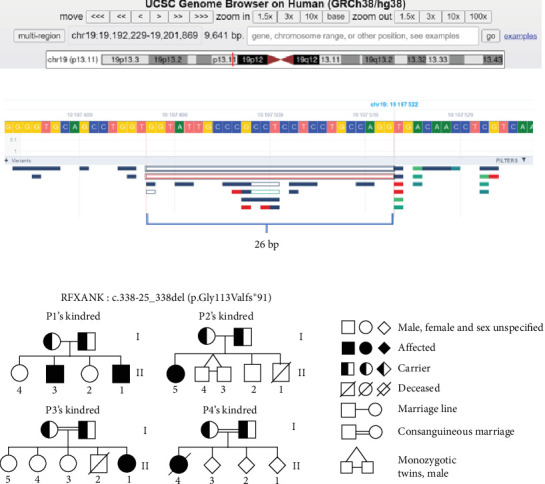
Genetic analysis and pedigree charts of Moroccan patients with MHC-II deficiency. (A) UCSC Genome Browser showing the location of the RFXANK gene on chromosome 19p13.11. (B) Detailed view of the c.338-25_338del mutation (p.Gly113Valfs*⁣*^*∗*^91) and its 26 bp deletion encompassing the intron 5/exon 6 boundary in the *RFXANK* gene. (C) Pedigree charts of the kindreds of patients with MHC-II deficiency, illustrating the autosomal recessive inheritance pattern. For patient P4, the sex of siblings is not specified due to missing data.

A chest X-ray showed thoracic expansion. Thoracic computed tomography (CT) revealed cylindrical bronchiectasis in the left lobe with mucosal impaction. (Figure 1). Sputum cytobacteriological examinations identified *Hemophilus influenzae*, *Pneumococcus*, and *Staphylococcus* (Table 2). Cystic fibrosis was considered but ruled out based on sweat test result (56 mmol/L). HIV serology was performed and was negative. Blood cell count showed a regenerative microcytic hypochromic anemia, from which he eventually recovered. Lymphocyte population studies revealed CD4 lymphopenia, when compared to age-matched reference values [20], with a low expression of HLA class II on B lymphocyte (2.4%), confirming the diagnosis of MHC-II deficiency (Table 3). Genetic analysis identified a homozygous mutation of *RFXANK* on chromosome 19p13.11, with a 26 bp deletion (c.338-25_338del; (Figure 2). Unfortunately, the diagnosis was delayed until the age of 15 years.

Following the diagnosis, I.S. is under consideration for HSCT. In the meantime, he is receiving respiratory physiotherapy and nutritional support, but immunoglobulin substitution therapy has not been initiated.

P2: A.K. is a 16-year-old female adolescent, the youngest of five siblings, born to nonconsanguineous parents. She has a notable familial history, as one of her brothers passed away at the age of 4 months due to cardiac arrest after multiple hospitalizations for recurrent respiratory tract infections without a definitive diagnosis.

**Figure 3:**
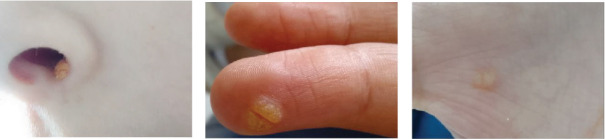
Warts located on the nose (A), right index finger (B), and palm of the right hand (C) of patient 2.

A.K.'s symptoms began at the age of 3 months with recurrent respiratory infections. By the age of 3 years, she had been hospitalized multiple times for recurrent pneumonia, otitis media, and chronic sinusitis. She also presented with failure to thrive during her early childhood. Upon admission to our department at the age of 14 years, A.K. was diagnosed with superinfected bronchiectasis. Physical examination revealed multiple warts localized on her nose, right index finger, and the palm of her left hand (Figure 3). A thoracic CT scan demonstrated bilateral cylindrical and moniliform bronchiectasis associated with a middle lobar condensation focus and mediastinal adenopathies (Figure 1A, a3, a4; B, b2–b5). A sinus CT scan revealed pansinusitis with a polypoid aspect of the sphenoidal sinus (Figure 1A, a5).

Laboratory workup revealed mild microcytic anemia without quantitative leukocyte abnormalities on the CBC. Sputum cytobacteriological examinations identified alpha-hemolytic *Streptococcus* and *Pseudomonas aeruginosa*. Total immunoglobulin levels were measured, showing normal IgG and IgA levels, but a selective decrease in IgM was detected. Immunophenotyping showed a moderate CD4+ lymphopenia associated with NK cell lymphopenia. HLA-DR expression was markedly decreased, confirming the diagnosis of MHC II deficiency. Genetic analysis identified the c.338-25_338del mutation in the associated gene (Figure 2).

Currently, A.K. is receiving symptomatic treatment, including monthly intravenous immunoglobulin (IVIG) substitution, physiotherapy, nutritional support, and antibiotic prophylaxis with cotrimoxazole and azithromycin. Her care plan emphasizes aggressive treatment of infections to prevent further complications.

P3: A.A., a 17-year-old female patient, the eldest of five siblings, born to first-degree consanguineous parents. She has a significant familial history, including the death of a brother at the age of seven due to an undiagnosed infectious condition.

A.A. was first admitted to our department at the age of 3 months with symptoms of vomiting, chronic diarrhea, and prolonged fever. Over time, she experienced multiple hospitalizations for recurrent fever and episodes of diarrhea. Although initial stool cultures and parasitological tests were negative, subsequent targeted investigations revealed the presence of *Entamoeba histolytica* and *Candida albicans*. A.A. also exhibited psychomotor impairment and had a family history of atopy.

Her blood count showed moderate microcytic anemia at 8.7 g/dL without leukocyte abnormalities (Table 3). Immunoglobulin measurements revealed an isolated decrease in serum IgA levels. Immunophenotyping showed a normal CD4+ T lymphocyte count for her age, associated with NK lymphopenia. She was diagnosed with MHC-II deficiency at the age of 15 months based on the absence of HLA-DR expression on CD19+ lymphocytes.

Following her diagnosis, A.A. was started on monthly IVIG therapy and antibiotic prophylaxis. However, she developed bronchiectasis and continues to experience occasional infectious episodes, which are treated with antibiotics. Additionally, she has shown failure to thrive.

Currently, A.A. remains on monthly IVIG therapy and continues to be monitored for infectious complications and growth development.

P4: C.E. is a 26-year-old female, the youngest of four siblings, with an indication of 1st-degree consanguinity. She has a history of recurrent respiratory tract infections, oral thrush, and recurrent oral aphthosis during childhood. At the age of 15 years, she underwent surgery for undocumented gynecological problems.

At the age of 23, C.E. was diagnosed with ulcerative colitis and was started on full-dose corticosteroid therapy. Subsequently, she developed disturbed liver function, diaphragmatic cupola hernia pain, and a multidrug-resistant respiratory infection.

On physical examination, her skin, mucosa, and integument revealed a moon face appearance, with a polycyclic erosion of the lower lip showing a yellowish background and multiple erosions of the gums and palate with regular and friable contours. On the anterior side of her left arm, she had a polycyclic, snail-like erosion, and under the left little finger, an eroded papule with regular contours.

Magnetic resonance imaging (MRI) revealed inflammatory stenosis. X-ray and chest CT scans showed an alveolar-interstitial syndrome, moderate dilatation of intrahepatic biliary ducts, discrete inflammatory-type rectal thickening, and a focus of bronchiectasis in the middle lobe. Viral serologies for HIV, CMV, and HCV were negative. Screening for antinuclear antibodies demonstrated a fine granular nuclear and cytoplasmic pattern with a titer of 640 with no specific antinuclear antibody identified.

A suspicion of primary immunodeficiency was raised after ruling out systemic lupus erythematosus. Complete blood count revealed lymphopenia at 555/mm³. Analysis of lymphocyte subpopulations showed lymphopenia of T CD4+, B, and NK cells, with an increase in T CD8+ lymphocytes. Immunoglobulin levels did not reveal hypogammaglobulinemia (Table 3). HLA-DR expression was absent (0%), confirming a diagnosis of MHC-II deficiency. Genetic analysis identified the same mutation reported in three previously documented cases. Despite therapeutic interventions, including supportive care, C.E. passed away a few months after the diagnosis.

### 3.2. Cases From the Literature

Eight patients aged 15 years or older with MHC-II deficiency has been reported in the literature, including four of Algerian. Detailed demographic and clinical data are presented in Table 4.

## 4. Discussion

MHC-II deficiency is associated with a poor prognosis, with most patients having a life expectancy of just a few years [21]. The average survival reported in the literature is generally very short. Most children succumb to severe or opportunistic infections within the first years of life, often before the age of 4 or 5 [18]. Our study included three females and one male, all unrelated, with a mean age of 18.75 years (range 16–26 years), and two (50%) with an indication of consanguinity. The mean age at onset of symptoms and at diagnosis were 5.25 months and 14.07 years, respectively. Of the eight patients with MHC-II deficiency reported in the literature from Algeria, Greece, and Iran, the mean age was 24.5 years (range 16–41 years), with a consanguinity rate of 62.5% (Table 4). All of our patients exhibited failure to thrive despite treatment. Three patients (75%) had bronchiectasis, with two receiving physiotherapy and nutritional management. All patients in our study and the literature received antibiotic prophylaxis and monthly immunoglobulin infusions. One of our patients, diagnosed late at 26 years, had severe complications including hemorrhagic rectocolitis and a multidrug-resistant respiratory infection, while another (25%) had a cow's milk protein allergy. In the literature, 100% of patients had pneumopathy and failure to thrive, 50% had had ear, nose, and throat infections, and some had liver disease, gastroenteritis, and autoimmune hemolytic anemia. One patient was positive for antineutrophil cytoplasmic antibodies. In our study, one patient tested positive for antinuclear antibodies.

In lymphocytes populations study, TCD4+ and CD16+/56 lymphopenia were observed in three of our patients (3/4). However, B lymphocyte counts were normal in three of the four tested patients (Table 5). In the literature review, all tested patients (3/4) had normal TCD4+ lymphocytes and increased TCD8+ lymphocytes. These results highlight the variability in lymphocyte subpopulation profiles observed among patients. Even TCD4 lymphopenia, which is a characteristic feature of MHC-II deficiency [18], is not a consistent criterion. Residual expression of MHC-II molecules on B lymphocytes or monocytes has been proposed to explain the mild phenotypes. Notably, Wiszniewski et al. [19] demonstrated low HLA-DR, DQ, and DP expression on B cells and monocytes. In our study, residual expression does not appear to be critical to the outcome, as it was not detected (<5%) in the B lymphocytes or monocytes of any of our patients. In addition, Ben Mustapha et al. [22] reported eight patients with residual MHC-II molecules who had a fatal outcome. Other genetic and environmental factors may explain the variable clinical expression. From our literature review, we did not consider the lymphocyte subpopulation and immunoglobulin results of four patients, as it was impossible to distinguish them from the 35 patients reported by Ouederni et al. [18].

The MHC-II genes are located on the short arm of chromosome 6 [23, 24]. Several studies have shown that the loci and promoters of these MHC-II genes are normal, and the pathology is caused by mutations in four transcriptional regulatory factors [25–28]. Among these factors, mutations in *RFXANK* account for 70% of MHC-II deficiencies, with 19 mutations identified to date. The most common mutation in North Africa and the Middle East is the c.338-25_338del26 mutation [16, 22, 29–31], likely due to a founder effect that occurred in this population ~2250 years ago [16, 18]. This mutation, which is a 26 bp deletion encompassing the intron 5/exon 6 boundary in the *RFXANK* gene, was detected in all our patients (*n* = 4; 100%). A previous study reported the same homozygous mutation in four patients (50%) [18]. However, another homozygous splice-site mutation (c.438+5G >A) [17] in *RFXANK* has been documented only in one patient (12.5%) (Table 6). Moreover, CIITA was responsible for this pathology in three cases in the literature review [19]. This is a homozygous T1524C mutation causing a substitution of leucine for proline at position 469 (L469P). The implication of this gene seems to be highly widespread in European countries, with ~16 mutations reported [32]. In the three patients of Greek origin reported in the literature review, this missense mutation prevented normal MHC-II function but did not lead to nuclear exclusion of L469P CIITA. Transfection experiments have demonstrated that the CIITA L469P mutation has residual activity, which could explain milder infections and, consequently, survival beyond early childhood [19].

While HSCT is the only curative treatment for MHC-II deficiency [33], many patients in resource-limited settings remain on lifelong prophylactic treatments due to the unavailability of suitable donors or transplantation options. Three of our patients are still alive. The surviving patients are regularly treated with immunoglobulins and antibiotic prophylaxis with trimethoprim-sulfamethoxazole. However, there appears to be significant variability in the outcome of patients with MHC-II deficiencies. The 42-year-old Iranian patient, diagnosed late at the age of 18 years, developed complications such as autoimmune hemolytic anemia, and positive antineutrophil cytoplasmic antibodies requiring azathioprine treatment at the age of 36 years, as well as recurrent urinary tract infections. Despite these complications, she remains alive thanks to immunoglobulin treatment [17]. One of our patients, P3, was diagnosed with MHC-II deficiency at the age of 15 months, following several hospitalizations for infectious episodes. Since the age of 3 years, her clinical course has been stable. While she continues to receive monthly IVIG treatment, her infectious episodes have been managed on an outpatient basis without the need for hospitalization, highlighting the possibility of mild presentations in some patients. These cases underscore the diversity of clinical presentations and emphasize the importance of early and appropriate management of patients with MHC-II deficiency to improve their prognosis and life expectancy.

## 5. Conclusion

It is important to consider MHC-II deficiency in the differential diagnosis of combined immunodeficiencies across all age groups. Further studies are needed to understand the various phenotypes associated with this condition. The only curative treatment is bone marrow transplantation. However, early diagnosis and management with IVIG can aid in extending the lifespan of affected individuals.

## Figures and Tables

**Table 1:** Studies of major histocompatibility complex (MHC) class II deficiency included in our literature review.

References	Number of patients^a^	Patients meeting our criteria^b^
Khorchidi et al. [17]	18	1 (P10)
Ouederni et al. [18]	35	4 (P6, P8, P14, P35)
Wiszniewski et al. [19]	3	3 (SaE, SaM, SaA)

Total	56	8

^a^Total number of patients incuded in each series.

^b^Patients included in the literature review.

**Table 2:** Clinical details of the four study patients.

	P1 (I.S.)	P2 (A.K.)	P3 (A.A.)	P4 (C.E.)
Sex	M	F	F	F
Age (years)	16	16	17	26
Age of onset of symptoms	11 months	3 months	1 month	6 months
Age at time of diagnosis	15 years	14 years	15 months	26 years
Consanguinity	No	No	1st degree	1st degree
Respiratory tract infections	Yes	Yes	Yes	Yes
Prolonged diarrhea	Yes	Yes	Yes	Yes
Urinary infection	Yes	No	No	Yes
Mucous membrane infection	No	Yes	No	Yes
Failure to thrive	Yes	Yes	Yes	Yes
Other expressions	Bronchiectasis Cow's milk protein allergy Meningitis	Bronchiectasis Warts	None	Bronchiectasis Hemorrhagic rectocolitis
Isolated microbes	*Hemophilus influenzae* ^a^ *Pneumococcus* ^a^ *Staphylococcus* ^a^	Alpha-hemolytic *Streptococcus*^a^ *Pseudomonas*^a^	*Entamoeba histolytica* ^b^ *Candida albicans* ^b^	—
Treatment	Physiotherapy	Physiotherapy, antibiotic prophylaxis, nutritional management, and monthly IVIG infusion	Monthly IVIG infusion	—
Progress	Alive	Alive	Alive	Deceased

Abbreviations: F, Female; IVIG, Intravenous immunoglobulin; M, Male.

^a^Sputum sample.

^b^Stool sample.

**Table 3:** Paraclinical findings in study patients.

Paraclinical examinations	P1 (I.S.)	P2 (A.K.)	P3 (A.A.)	P4 (C.E.)
Blood count				
Hemoglobin (g/dL)	**10.2** (12.5–16.0)	**11** (12.5–16.0)	**8.7** (12.5–16.0)	12.5 (12.5–16.0)
MCV (μm³)	**72.9** (79–96)	**65** (79–96)	**70.4** (79–96)	84 (79–96)
MCHC (g/dL)	**27.3** (28–36)	32 (28–36)	32.6 (28–36)	32 (28–36)
MCH (pg)	**19.9** (27–33)	**17.4** (27–33)	**23** (27–33)	ND
White blood cells (/mm³)	9720 (4500–11,400)	10,700 (4500–11400)	10,120 (4500–11,400)	7620 (4500–11,400)
Lymphocytes (/mm³)	1790 (>1200)	5778 (>1200)	3910 (>1200)	**555** (>1200)
Polymorphonuclear neutrophils (/mm³)	7020 (2000–7500)	3852 (2000–7500)	ND	6620 (2000–7500)
Polymorphonuclear basophils (/mm³)	0.03 (0–600)	0	ND	ND
Polymorphonuclear eosinophils (/mm³)	0.15 (0–100)	0	ND	ND
Platelets (/mm³)	**525,000** (160–385,000)	306,000 (160–385,000)	364,000 (160–385,000)	348,000 (160–385,000)
**Immunoglobulins (g/L)**				
IgA	ND (0.50–2.00)	0.58 (0.50–2.00)	**0.25** (0.70–3.40)	1.09 (0.50–2.00)
IgM	ND (0.50–1.60)	**0.13** (0.50–1.60)	1.01 (0.50–2.10)	1.53 (0.50–1.60)
IgG	ND (6.50–12.30)	6.66 (6.50–12.30)	7.58 (6.60–12.80)	9.08 (6.50–12.30)
IgG1	ND	3.55 (3.15–11.5)	ND	ND
IgG2	ND	**0.446** (0.64–4.95)	ND	ND
IgG3	ND	0.553 (0.23–1.96)	ND	ND
IgG4	ND	**0.003** (0.037–1.57)	ND	ND
IgE (IU/mL)	ND	ND	ND	ND
Lymphocyte subsets (/mm³)				
Total lymphocytes	2300	2800	3010	1436
CD3+ (/mm³)	1914 (1000–2200)	2575 (1000–2200)	**2663** (1000–2200)	1367 (967–1725)
CD3+CD4+ (/mm³)	**419** (530–1300)	**481** (530–1300)	863 (530–1300)	**50** (507–955)
CD3+CD8+ (/mm³)	**1240** (330–920)	**1726** (330–920)	**1650** (330–920)	**1152** (404–826)
CD3-CD19+ (/mm³)	242 (110–570)	167 (110–570)	638 (330–920)	**43** (47–243)
CD16+/CD56+ (/mm³)	99 (70–480)	**2** (70–480)	**38** (70–480)	**23** (94–348)
HLA-DR+/CD19+	**2.4** (<5%)	**0.4** (<5%)	**00** (<5%)	**00** (<5%)
Serology				
HIV	Negative	Negative	Negative	Negative
CMV	Negative	Negative	**Positive**	Negative
Aspergillosis serology	Negative	Negative	Negative	Negative
Specific IgE aspergillus (ABPA)	Negative	Negative	Negative	Negative
Other examinations				
Antinuclear antibody testing	ND	ND	ND	**1/640** (≤1/80)

*Note:* Abnormal values are in bold. Reference values by age according to Shearer et al. [20].

Abbreviations: ND, not done; NR, normal range.

**Table 4:** Demographic and clinical information of patients with major histocompatibility complex (MHC) class II deficiency in our study and from the literature review.

	Our patients (*n*; %)	Patients from the literature (*n*; %)
Origin	Moroccan (4; 100%)	Algerian (4; 50%) [18], Greek (3; 37.5%) [19], and Iranian (1; 12.5%) [17]
Female	3 (75)	4 (50)
Male	1 (25)	4 (50)
Mean age (range), (years)	18.75 (16–26)	24.5
Mean age at onset (months)	5.25	6.25
Mean age at diagnosis (years)	14.07	13.4
Consanguinity	2 (50)	5 (62.5)
Failure to thrive	4 (100)	8 (100)
Pneumonia	4 (100)	8 (100)
Chronic gastroenteritis/diarrhea	3 (75)	3 (37.5)
Bronchiectasis	3 (75)	2 (25)
ENT infection	2 (50)	4 (50)
Urinary tract infection	2 (50)	1 (12.5)
Mucosal skin infection	2 (50)	0
Sinusitis	2 (50)	1 (12.5)
Liver disease	1 (25)	3 (37.5)
Autoimmune hemolytic anemia (AHIA)	1 (25)	3 (37.5)
Antineutrophil cytoplasmic antibodies (ANCA)	0	1 (12.5)
Antinuclear and fine granular antibodies (ANA)	1 (25)	0
Meningitis	1 (25)	1 (12.5)
Asthma	0	1 (12.5)
Cholestasis	0	1 (12.5)
Septicemia	0	1 (12.5)
Sepsis	0	1 (12.5)
Isolated germs *N* (%)		
* Haemophilus influenzae*	1 (25)	3 (37.5)
Alpha-hemolytic *Streptoccocus*	1 (25)	0
* Streptoccocus pneumoniae*	0	2 (25)
* Staphylococcus*	1 (25)	0
* Pneumococcus*	1 (25)	0
Cytomegalovirus	1 (25)	2 (25)
Herpes simplex virus	0	2 (25)
Varicella	0	2 (25)
* Campylobacter jejuni*	0	2 (25)
*Salmonella enteritidis*	0	2 (25)
* Klebsiella pneumoniae*	0	1 (12.5)
* Listeria monocytogenes*	0	1 (12.5)
* Helicobacter pylori*	0	1 (12.5)
* Cryptosporidium parvum*	0	1 (12.5)
* Clostridioides difficile*	0	1 (12.5)
* Candida albicans*	1 (25)	0
* Entamoeba histolytica*	1 (25)	0
Treatment (*n*, %)	Antibiotic prophylaxis Monthly IVIG infusion (4, 100)	Antibiotic prophylaxis Monthly IVIG infusion (8, 100)
Deceased (*n*, %)	1 (25)	1 (12.5)

Abbreviation: IVIG, intravenous immunoglobulin.

**Table 5:** Summary of immunophenotyping of patients with major histocompatibility complex (MHC) class II deficiency in our study and from the literature review.

Immunophenotyping	Our patients (*N* = 4)	Literature review patients (*N* = 4)	Total (*n/N*)
CD3+ lymphopenia	2	0	2/8
CD3+CD4+ lymphopenia	3	1	4/8
CD3+CD8+ lymphopenia	0	0	0/8
CD19+ lymphopenia	1	0	1/8
CD16+/56+ lymphopenia	3	1	4/8
HLA-DR expression < 5%	4	4	8/8

**Table 6:** Mutations responsible for major histocompatibility complex (MHC) class II deficiency in patients in our study and from the literature review.

	Our patients	Literature review patients		
Number	4	4 [18]	1 [17]	3 [19]
Gene	RFXANK	RFXANK	RFXANK	CIITA
Chromosomal location	19p13.11	19p13.11	19p13.11	16p13.13
Mutation	c.338-25_338del	c.338-25_338del	c.438+5G >A	T1524C
Zygosity	Homozygote	Homozygote	Homozygote	Homozygote

*Note*: RFXANK, X regulatory factor containing ankyrin repeats.

Abbreviation: CIITA, class II transactivator.

## Data Availability

The data that support the findings of this study are available from the corresponding author upon reasonable request.
